# Association of HFE Gene Mutations With Liver Cirrhosis Depends on Induction of Iron Homeostasis Disturbances

**DOI:** 10.5812/hepatmon.825

**Published:** 2012-03-28

**Authors:** Katarzyna Sikorska

**Affiliations:** 1 Department of Infectious Diseases, Medical University of Gdansk, Smoluchowskiego, Gdansk, Poland

**Keywords:** Genes, Homeostasis, Liver Cirrhosis

Dear Editor,

I read with the interest the paper by Jowkar et al. published in a recent issue of Hepatitis Monthly [1]. The authors analysed the frequency of two HFE gene mutations in Iranian patients with a diagnosis of cryptogenic cirrhosis. In Europe, North America and Australia the homozygous C282Y mutation of the HFE gene is a major etiological factor associated with the pathogenesis of progressive iron accumulation leading to multiorgan disfunction, as it is observed in hereditary hemochromatosis. About 5% of hereditary hemochromatosis cases in the Caucasian population are related to compound heterozygosity for C282Y and H63D mutations [2]. The impact of other HFE gene mutations on the development of iron overload is discussed [3]. Despite the relatively high frequency of HFE gene mutations among Caucasians, their phenotypic expression are limited and the causes of this are not fully identified. Moreover among Caucasians, the frequency of HFE gene mutated alleles is clearly differentiated, which determines the real significance of the mutations in the development of genetically determined iron overload [2]. In Polish patients with a diagnosis of liver cirrhosis, iron overload symptoms were observed in 70% of cases and in this selected population the homozygous C282Y mutation appeared to occur more frequently in comparison to the healthy population [4]. Jowkar et al. did not confirm the association of HFE gene mutations with a diagnosis of cryptogenic cirrhosis in Iranian patients, which seems to lead to the erroneous conclusion that the HFE gene defect has no pathogenic significance. The authors included mainly patients without symptoms of iron overload (86%) in their study. They also did not describe how they assessed the patients' iron status. The recruitment of only cryptogenic cirrhosis cases means that iron overload patients suspected of hereditary hemochromatosis were excluded. Thus the results of the study are not surprising, however they cannot serve as a complete source of information about the influence of the HFE gene mutations on the development of liver cirrhosis. In my opinion, in the discussion concerning the potential influence of HFE gene mutations on morbidity, e.g. course of liver diseases, analysis of their occurrence should always be related to the presence of iron metabolism disturbances. An undeniable fact is that the increased risk of liver diseases (hepatocellular carcinoma, hepatitis C, non-alcoholic steatohepatitis) that was confirmed in carriers of C282Y mutations, is a result of disturbed iron homeostasis caused by HFE protein dysfunction [5].
